# Distinguishing Clinical *Enterococcus faecium* Strains and Resistance to Vancomycin Using a Simple In-House Screening Test

**DOI:** 10.3390/antibiotics11030286

**Published:** 2022-02-22

**Authors:** Natkamon Saenhom, Parichart Boueroy, Peechanika Chopjitt, Rujirat Hatrongjit, Anusak Kerdsin

**Affiliations:** 1 Faculty of Public Health, Kasetsart University Chalermphrakiat Sakon Nakhon Province Campus, Sakon Nakhon 47000, Thailand; natkamon030916@gmail.com (N.S.); parichart.bou@ku.th (P.B.); peechanika.c@ku.th (P.C.); 2 Faculty of Science and Engineering, Kasetsart University Chalermphrakiat Sakon Nakhon Province Campus, Sakon Nakhon 47000, Thailand; Rujirat.ha@ku.th

**Keywords:** Enterococci, *Enterococcus faecium*, screening test, vancomycin

## Abstract

Vancomycin-resistant enterococci (VRE) are a major concern as microorganisms with antimicrobial resistance and as a public health threat contributing significantly to morbidity, mortality, and socio-economic costs. Among VREs, vancomycin-resistant *Enterococcus faecium* (VREfm) is frequently isolated and is resistant to many antibiotics used to treat patients with hospital-acquired infection. Accurate and rapid detection of VREfm results in effective antimicrobial therapy, immediate patient isolation, dissemination control, and appropriate disinfection measures. An in-house VREfm screening broth was developed and compared to the broth microdilution method and multiplex polymerase chain reaction for the detection of 105 enterococci, including 81 VRE isolates (61 *E. faecium*, 5 *E. faecalis*, 10 *E. gallinarum*, and 5 *E. casseliflavus*). Verification of this screening broth on 61 VREfm, 20 other VRE, and 24 non-VRE revealed greater validity for VREfm detection. The accuracy of this broth was 100% in distinguishing *E. faecium* from other enterococcal species. Our test revealed 93.3% accuracy, 97.5% sensitivity, and 79.2% specificity compared with broth microdilution and PCR detecting *van* genes. The kappa statistic to test interrater reliability was 0.8, revealing substantial agreement for this screening test to the broth microdilution method. In addition, the in-house VREfm screening broth produced rapid positivity after at least 8 h of incubation. Application of this assay to screen VREfm should be useful in clinical laboratories and hospital infection control units.

## 1. Introduction

Enterococci are known as opportunistic pathogens and a leading cause of hospital-acquired infections, especially vancomycin-resistant enterococci (VRE) that are a major concern regarding their antimicrobial resistance as a public health issue. The two major species that are well-known worldwide to cause human diseases and resistance to vancomycin are *Enterococcus faecium* and *E. faecalis* [1]. Of these two species, vancomycin-resistant *E. faecium* (VREfm) is more frequently isolated from patients with hospital-acquired infection than vancomycin-resistant *E. faecalis* and other species [1].

The World Health Organization (WHO) published their list of priority bacterial pathogens for which new antibiotics are urgently needed, and VREfm is listed in the high-priority category of antibiotic-resistant bacteria [2]. VREfm is widely distributed in hospitals around the world, with the prevalence varying according to geographical location. In the U.S., VREfm accounts for 82% of reported cases because vancomycin is rarely restricted and thus has widespread antibiotic use in hospitals [3,4,5]. In European countries, VREfm has shown increasing prevalence, from 10% in 2015 to 17.3% in 2018 [3]. In Asia, the prevalence of VREfm accounts for 22.4% of reported cases and is higher than for European countries but lower than for the U.S. [6]. Treatment options for invasive VREfm infections are very limited, resulting in high mortality [7]. Vancomycin resistance determinants due to the *vanA* and *vanB* genes are globally frequently reported in VRE, including in *E. faecium* clinical isolates [3,8].

A previous report discussed potential determinants influencing the future dissemination and control of antibiotic resistance and nominated the rapidity and accuracy of laboratory techniques that allowed for rapid identification of the infecting pathogen and antibiotic susceptibility testing [9]. Therefore, accurate and rapid detection of VRE, especially VREfm, support effective antimicrobial therapy, immediate patient isolation, and appropriate disinfection measures in hospitals. Many methods have been implemented to identify either VRE or VREfm, including conventional culture, real-time PCR, conventional PCR, and automated microbiology instruments, such as BD Phoenix (Becton, Dickinson and Company) or Vitek-2 (bioMerieux) [10,11]. Although some of these methods are rapid, easy to perform, and are accurate in identification or detection, many of these methods are expensive and have a high level of sophistication that requires operator experience and skill and special instrumentation. These constraints make such methods inappropriate in a laboratory with limited resources. Therefore, we developed an alternative assay to detect VREfm isolates at a low cost. The aim of this study was to compare an in-house VREfm screening broth with the broth microdilution method and multiplex PCR for the detection of VREfm. Our screening broth provides an alternative assay to detect VREfm isolates. This may be useful in laboratories where a large number of isolates must be screened at low cost and as such could reduce the costs associated with laboratory and infection control in hospitals with a high prevalence of VRE, especially VREfm. 

## 2. Results

In this study, we compared the in-house VREfm screening broth using the broth microdilution method and multiplex PCR for the detection of VREfm. All tested enterococci were confirmed to the species level, *van* genes detected, and the MIC values determined of vancomycin. The screening broth was evaluated using the broth microdilution method and PCR assay in terms of specificity, sensitivity, accuracy, and Cohen’s kappa coefficient.

### 2.1. Identification and TestingVvancomycin-Resistant Strains 

**Figure 1:**
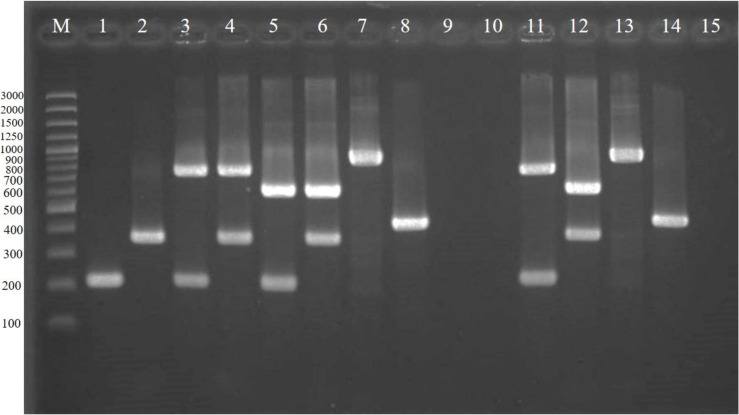
Agarose gel electrophoresis of PCR-amplified products from local enterococci (lane 1–10) and reference enterococci (lane 11–14). *E. faecium* (lane 1), *E. faecalis* (lane 2), *E. faecium* carrying *vanA* (lane 3), *E. faecalis* carrying *vanA* (lane 4), *E. faecium* carrying *vanB* (lane 5), *E. faecalis* carrying *vanB* (lane 6), *E. gallinarum* carrying *vanC1* (lane 7), *E. casseliflavus* carrying *vanC2/C3* (lane 8), *E. muntdii* (lane 9), *E. raffinosus* (lane 10), *E. faecium* ATCC BAA-2316 carrying *vanA* (lane 11), *E. faecalis* ATCC51299 carrying *vanB* (lane 12), *E. gallinarum* ATCC49608 carrying *vanC1* (lane 13), *E. casseliflavus* ATCC700668 carrying *vanC2/C3* (lane 14), and blank control (lane 15). A 100-bp DNA ladder is shown in lane M.

As shown in Table 1, among 105 enterococci confirmed using conventional biochemical tests and multiplex PCR (Figure 1), 71 were *E. faecium* isolates and 14 were *E. faecalis* isolates. The 20 *Enterococcus* spp. identified using conventional biochemical tests were *E. gallinarum* (*n* = 10), *E. casseliflavus* (*n* = 5), *E. mundtii* (*n* = 4), and *E. raffinosus* (*n* = 1). The multiplex PCR assay also detected *vanA* in 51 out of the 105 enterococci, consisting of 48 *E. faecium* and 3 *E. faecalis* isolates, respectively, whereas *vanB* were detected in 15 isolates, consisting of 13 *E. faecium* and 2 *E. faecalis* isolates. We detected *vanC1* in all *E. gallinarum* and *vanC2/C3* in all *E. casseliflavus*, whereas *vanA*, *vanB*, *vanC1*, or *vanC2/C3* genes were not detected in any of the *E. mundtii* and *E. raffinosus* samples.

The vancomycin MIC values indicated that 85.9% (61/71) of the *E. faecium* isolates were resistant to vancomycin (range 32–128 μg/mL) that were confirmed to be VRE according to their MIC values and the presence of either *vanA* or *vanB*, whereas the remaining 10 isolates were susceptible to vancomycin. Of the 14 *E. faecalis* isolates, only five were resistant to vancomycin (VREfs) by broth microdilution, with 3 isolates having an MIC value of 64 or 128 μg/mL, and they all carried *vanA*, while the remaining two *E. faecalis* isolates carrying *vanB* revealed an MIC value of 32 μg/mL. In contrast with the 20 *Enterococcus* spp., only 10 *E. gallinarum* and 5 *E. casseliflavus* isolates showed either intermediate or full resistance to vancomycin with MIC values in the range 8–64 μg/mL, whereas the rest were susceptible (vancomycin-susceptible enterococci, VSE). The results are summarized in Table 1.

### 2.2. Evaluation of In-House VREfm Screening Broth

**Figure 2:**
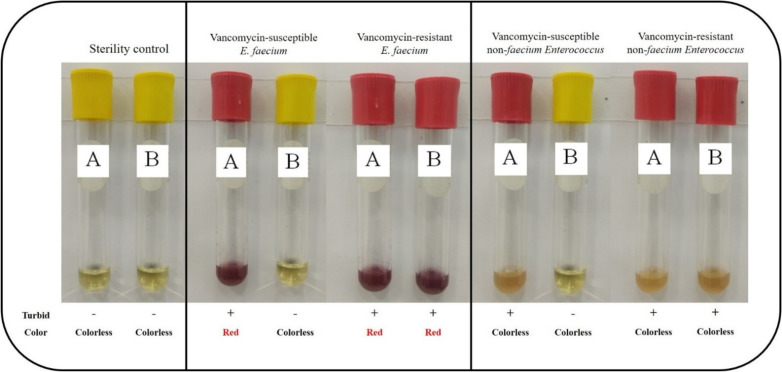
Interpretation of in-house VREfm screening broth for vancomycin-resistant enterococci (VRE) and vancomycin-susceptible enterococci (VSE).

The in-house VREfm screening broth determined resistance to vancomycin based on turbidity and distinguished *E. faecium* from other enterococci based on color changes. The broth was used in two tubes (A and B). Tube A did not contain vancomycin, whereas tube B did (6 μg/mL vancomycin). Basically, all enterococci, including those that were vancomycin not-susceptible or susceptible, could grow in tube A, while growth in tube B depended on their resistance to the antibiotic. *E. faecium* could produce β-galactosidase, the enzyme degraded to Salmon-Gal that produced a red product, whereas the other *Enterococcus* spp. did not (Figure 2). 

We verified this screening broth using broth microdilution as a reference method on 105 enterococci. These included 61 VREfm, 5 VREfs, 4 vancomycin-resistant *E. gallinarum* (VREg), and one vancomycin-resistant *E. casseliflavus* (VREc), while the other 34 isolates consisted of vancomycin-intermediate isolates (6 *E. gallinarum*, VIEg; and 4 *E. casseliflavus*, VIEc) and vancomycin-susceptible *E. faecalis* (VSEfs; *n* = 9), *E. faecium* (VSEfm; *n* = 10), *E. mundtii* (VSEm; *n* = 4), and *E. raffinosus* (VSEr; *n* = 1). As shown in Table 1, all VREfm showed turbidity and the presence of red color in both tubes. The other VREs (VREfs, VREg, VREc) showed turbidity but with no color in either tube. 

We found 5 VSEs (2 were VSEfm and 3 were VSEfs) that produced a positive result in tube B (containing vancomycin), indicating that they were vancomycin-resistant based on the screening broth. Indeed, they were susceptible to vancomycin based on broth microdilution with an MIC value of 4 μg/mL for all isolates (Table 1). In contrast, one isolate each of VIEg and VIEc showed neither turbidity nor color in tube B, although they showed intermediate resistance to vancomycin with an MIC value of 8 μg/mL (Table 1). This suggested that our test had variation with the borderline MIC cut-off for some intermediate isolates, especially for VIEg or VIEc that carried *vanC*. However, there was good correlation of our screening broth and broth microdilution to indicate vancomycin resistance at MIC ≥16 μg/mL. 

The screening broth could identify *E. faecium* by producing the red color in all 71 isolates, while all the other species of *Enterococcus* (*n* = 34) were colorless. This demonstrated that the accuracy of this broth-based method was 100% for identification of *E. faecium* according to multiplex PCR assay. However, determination of vancomycin resistance and presence of *van* genes revealed 93.3% accuracy, 97.5% sensitivity, and 79.2% specificity (Table 2 and Table 3). The kappa statistic to test interrater reliability showed 0.8 for this screening test. Based on the kappa criterion, our in-house VREfm screening broth had substantial agreement (values in the range 0.61–0.80) to the broth microdilution and PCR detecting *van* genes as the reference method. In addition, our in-house VREfm screening broth showed rapid positivity after at least 8 h of incubation, and the cost per test (2 tubes) was USD 0.9 or EUR 0.8. 

## 3. Discussion

VREfm has a global impact as a threat to public health according to the WHO, and it contributes significantly to morbidity, mortality, and the socio-economic costs of healthcare-acquired infection [2,12]. For example, the daily costs of contact isolation (considering only gloves and gowns) for each VREfm patient in a ward and an intensive care unit (ICU) were USD 10.8 and USD 17.3, respectively [13]. With the mean duration of isolation of 25 and 41.5 days for the ward and the ICU, respectively, a total cost was estimated to be USD 270 and USD 718, respectively [13]. Another study revealed that the overall hospital costs of blood-stream infection were significantly higher in VREfm cases (EUR 80,465) compared to VSEfm (EUR 51,365) and VSEfs (EUR 31,122) cases [14]. Screening is recommended for patients at high risk of VRE colonization to prevent and control the transmission of VRE. Our study successfully developed an in-house VREfm screening test to distinguish VREfm from other VRE and VSE isolates.

Validity testing based on the kappa coefficient demonstrated that our screening broth had strong agreement in the determination of VRE (especially VREfm) but false-positive or false-negative results may occur in either VIEg/VIEc or VSE isolates with low MIC values of about 8 or 4 μg/mL, respectively. For the false-positives in 5 VSE with MIC at 4 μg/mL, the actual MIC may be 6 or 7 μg/mL because the broth microdilution technique applied two-fold dilution of vancomycin that started from 0.25 μg/mL and produced 0.5, 1, 2, 4, 8, 16, 32, 64, and 128 μg/mL. The gap between 4 and 8 μg/mL may have contained the exact MIC for these 5 false-positive VSE isolates according to our screening broth, which contained 6 μg/mL of vancomycin, and so allowed these 5 isolates to grow. However, the false-negatives involving VIEg and VIEc had MIC values of 8 μg/mL (Table 2) and were PCR-positive for *vanC* (Table 3) but did not grow in tube B of the screening broth, even though we repeated both the broth microdilution and screening tests. The explanation for this has not been elucidated but it may depend on the strain characteristics or the low concentration of inoculum used that retarded bacterial growth under marginal conditions.

Another study evaluated chromogenic agar to screen VRE based on the different colors of colonies for either VREfm or VREfs [15]. A false-positive was detected in the medium with non-enterococci, such as *Staphylococcus sciuri*, *Streptococcus mutans*, or other enterococci (*E. casseliflavus* and *E. raffinosus*) [15]. Cross-reactivity of our screening broth may have occurred with *S. sciuri*, as mentioned in another study [15]. Performing catalase and Gram-stain testing of presumptive VRE isolates should increase the specificity. Notably, our screening broth identified *E. faecium* based on degradation of 6-chloro-3-indoxyl-β-D-galactopyranoside by its β-galactosidase. This enzyme is also present in some species of enteroccocci, such as *E. malodoratus*, *E. saccharolyticus*, *E. villorum*, and *E. dispar*; however, these species have very low prevalence in hospitals, and their resistance to vancomycin is very rare [16,17]. To increase the efficiency and rapidity of VREfm screening among patients, the screening broth should be further studied and validated on clinical specimens directly. In addition, our screening broth has some limitations, including that it does not diagnose other pathogenic enterococci, such as *E. faecalis*, and does not specify the MIC. Furthermore, the broth could not be detected where there were strains resistant to teicoplanin only. Therefore, further development of this screening broth should be carried out to identify *E. faecalis*, *E. gallinarum*, or *E. casseliflavus*, to specify the MIC values, and to determine whether it is possible to detect teicoplanin-resistant strains.

Resistance to vancomycin in *enterococci* is mediated by the *van* genes, with *vanA*, *vanB*, *vanC*, *vanD*, *vanE*, *vanG*, *vanL*, *vanM*, and *vanN* having been identified to date; in particular, *vanA* and *vanB* are predominant worldwide [18]. In the current study, all clinical VREfm and VREfs carried either *vanA* or *vanB*, which conferred high resistance to vancomycin with an MIC range of 32–128 µg/mL. Most of them carried *vanA* (77.3%). Accordingly, the specific character of *vanA*-carrying enterococci showed high resistance to vancomycin (MIC ≥ 64 µg/mL) and teicoplanin (MIC ≥8 µg/mL) [19]. The high prevalence of *vanA*-harboring *E. faecium* or *E. faecalis* observed in the current study was similar to reports for China, Japan, Iran, the Netherlands, Germany, Italy, Australia, Tunisia, Brazil, and Canada [18,20,21,22,23,24,25,26,27,28].

The annual estimated laboratory costs were projected to be USD 19,074 for 6372 patients screened in a pediatric hospital between 2010 and 2014 in Turkey [13]. Therefore, screening priorities should be based on the prevalence of infection and the financial resources of the institution. The previous study revealed laboratory cost per specimen for rectal swab culture and PCR was USD 2.7 and USD 40.5, respectively [13]. The cost per test (2 tubes) for our assay was USD 0.9 or EUR 0.8, whereas the cost of the gold standard is about USD 2.7–3.0 or EUR 2.4–2.6. Application of this assay for VRE screening from pure culture should be useful for clinical laboratories and hospital infection control units where there is high prevalence of VRE to provide prompt information to facilitate the rapid control of VRE dissemination in hospitals and more rapid implementation of isolation precautions regarding VRE carriage or infection to other patients, as well as reducing the cost. 

## 4. Materials and Methods

### 4.1. Bacterial Strains 

In total, 105 enterococci, consisting of 71 *E. faecium*, 14 *E. faecalis*, and 20 other *Enterococcus* spp., were used in this study. These enterococci were isolated and sent by hospitals for further confirmation by the Public Health Microbiological Laboratory Service of the Faculty of Public Health, Kasetsart University Chalermphrakiat Sakon Nakhon province campus under the Emerging Antimicrobial Resistant Bacteria Surveillance Program (EARB). *E. faecium* ATCC BAA-2316 (*vanA*), *E. faecalis* ATCC51299 (*vanB*), *E. gallinarum* ATCC49608 (*vanC1*), and *E. casseliflavus* ATCC700668 (*vanC2/C3*) were used as controls in the multiplex PCR assay. *E. faecalis* ATCC29212 and *E. faecium* ATCC BAA-2316 were used as the control for the broth microdilution. In addition, *E. faecium* ATCC BAA-2316, *E. faecium* ATCC19434 and *E. faecalis* ATCC29212 were used for controls for the in-house VREfm screening broth.

### 4.2. Microbiological Analysis

Each isolate was cultured on sheep blood agar at 37 °C for 18 h and identified using a conventional biochemical test, including arabinose utilization, resistance to 6.5% NaCl, bile esculin degradation, and PYR (pyrrolidonyl *β*-naphthylamide) degradation [16], and multiplex PCR assay to simultaneously identify *E. faecium* and *E. faecalis*, as well as the vancomycin-resistant genes; *vanA*, *vanB*, *vanC1*, and *vanC2/C3*, that were developed in our laboratory [29,30]. Genomic DNA from all isolates was extracted using a NucleoSpin^®^ Tissue Kit (Macherey-Nagel, Germany) according to the manufacturer’s instructions. The PCR reaction mixtures contained 1X JumpStart^TM^ REDTaq^®^ ReadyMix^TM^ Reaction Mix (Sigma-Aldrich, MO, USA) and 0.4 µM of each primer pair (IDT, Singapore; Table 4). The following PCR thermocycling parameters were used: initial activation of DNA polymerase at 95 °C for 3 min; 30 cycles of denaturation at 95 °C for 30 s, primer annealing at 55 °C for 30 s, and extension at 72 °C for 1.15 min; with a final extension at 72 °C for 5 min. The PCR products were resolved using gel electrophoresis for 30 min on 2% agarose gels (Vivantis, Selangor, Malaysia) in 0.5× TBE buffer (Vivantis, Malaysia). The gels were stained with ethidium bromide (Sigma-Aldrich, MO, USA) and visualized under ultraviolet light using a GeneGenius Bioimaging System (SynGene, Maryland, USA). The sizes of the PCR products were determined by comparison with a molecular size standard (GeneRuler™ 100 bp Plus DNA ladder, Thermo Fisher Scientific, Waltham, MA, USA) as shown in Figure 1.

Susceptibility to vancomycin was performed to determine minimal inhibitory concentration (MIC) using broth microdilution according to the current CLSI guidelines [31]. A 0.5 McFarland standard suspension of the isolate was made from culture grown on Muller Hinton agar plates (BD BBL, Bergen County, NJ, USA). The interpretation of MIC followed the criteria in the CLSI 2021 guidelines, namely ≤4 mg/mL is susceptible, 8–16 mg/mL is intermediate, and ≥32 mg/mL indicates resistance [31]. Standard enterococci strains of *E. faecalis* ATCC 29212 (*van* absence) and *E. faecium* ATCC BAA-2316 (*vanA* presence) were used for quality control for the broth microdilution.

### 4.3. In-House VREfm Screening Broth

This screening broth was prepared using 2 tubes (A and B). Tube A (1 mL) contained 1.85% Brain Heart Infusion broth (Merck, Kenilworth, NJ, USA) and 0.2% 6-chloro-3-indoxyl-β-D-galactopyranoside (Salmon-Gal; GoldBio, St. Louis, MO, USA). This concentration of Salmon-Gal was selected experimentally based on our optimization (data not shown). Tube B (1 mL) consisted of the same ingredients as tube A and also contained vancomycin (Sigma-Aldrich, St. Louis, MO, USA) at 6 μg/mL according to the 2021 CLSI guideline recommendation for VRE screening. Interpretation is shown in Figure 2. A 0.1-mL amount of inoculum at a concentration of 0.5 McFarland was inoculated into the broth.

### 4.4. Statistical Analysis

Diagnostic measures were calculated, such as sensitivity, specificity, and accuracy of each test. The kappa statistic was calculated to evaluate the associations and levels of agreement of the data [32]. 

## 5. Conclusions

We successfully developed an in-house VREfm screening broth to distinguish *E. faecium* from other *Enterococcus* spp., and to determine resistance to vancomycin in a single assay. This alternative screening procedure for VREfm could be useful for hospital infection control.

## Figures and Tables

**Table 1:** Results of minimal inhibitory concentration by broth microdilution and in-house VREfm screening broth to susceptibility of vancomycin on 105 enterococci in this study.

Enterococci	Total	*van* Gene	MIC Value (μg/mL)	N	MIC Interpretation	In-House VREfm Screening Broth						Sensitivity	Specificity
						Tube A (No Vancomycin)			Tube B (Containing Vancomycin)				
						Turbidity	Color	N	Turbidity	Color	N		
*E. faecium* (*n* = 71)	48	*vanA*	128	41	Resistance	+	Red	41	+	Red	41	100%	80%
			64	7		+	Red	7	+	Red	7		
	13	*vanB*	64	9		+	Red	9	+	Red	9		
			32	4		+	Red	4	+	Red	4		
	10	none	4	5	Susceptible	+	Red	5	+	Red	2		
			2	3		+	Red	3	-				
			1	2		+	Red	2	-				
*E. faecalis* (*n* = 14)	3	*vanA*	128	1	Resistance	+	Colorless	1	+	Colorless	1	100%	66.6%
			64	2		+	Colorless	2	+	Colorless	2		
	2	*vanB*	32	2		+	Colorless	2	+	Colorless	2		
	9	none	4	4	Susceptible	+	Colorless	4	+	Colorless	3		
			2	2		+	Colorless	2	-				
			1	1		+	Colorless	1	-				
			0.5	2		+	Colorless	2	-				
*E. gallinarum* (*n* = 10)	10	*vanC1*	64	1	Resistance	+	Colorless	1	+	Colorless	1	90%	ND *
			32	3		+	Colorless	3	+	Colorless	3		
			16	2	Intermediate	+	Colorless	2	+	Colorless	2		
			8	4		+	Colorless	4	+	Colorless	3		
*E. casseliflavus* (*n* = 5)	5	*vanC2/C3*	32	1	Resistance	+	Colorless	1	+	Colorless	1	80%	ND *
			16	1	Intermediate	+	Colorless	1	+	Colorless	1		
			8	3		+	Colorless	3	+	Colorless	2		
*E. muntdii* (*n* = 4)	4	none	0.5	3	Susceptible	+	Colorless	3	-			ND *	100%
			0	1		+	Colorless	1	-				
*E. raffinosus* (*n* = 1)	1	none	0.25	1	Susceptible	+	Colorless	1	-			ND *	100%
**Total**	**105**			**105**				**105**			**84**		

* ND = No data because denominator is zero.

**Table 2:** Validity of in-house VREfm screening broth to determine vancomycin resistance compared with broth microdilution.

In-House Screening Broth	Broth Microdilution		Validity
	Positive (Vancomycin Not-Susceptible) *	Negative (Vancomycin Susceptible)	
Positive (vancomycin resistance)	79	5	Accuracy = 93.3%
Negative (vancomycin susceptible)	2	19	
**Validity**	Sensitivity = 97.5%	Specificity = 79.2%	

* Vancomycin not-susceptible = intermediate or fully resistant to vancomycin.

**Table 3:** Validity of in-house VREfm screening broth to determine vancomycin resistance compared with PCR detecting *van* genes.

In-House Screening Broth	PCR		Validity
	vanA, vanB, vanC1/C2/C3	None	
Positive (vancomycin resistance)	79	5	Accuracy = 93.3%
Negative (vancomycin susceptible)	2	19	
**Validity**	Sensitivity = 97.5%	Specificity = 79.2%	

**Table 4:** Primers used in multiplex PCR in current study.

Primer Name	Sequence (5′-3′)	Target	PCR Product Size (bp)	Reference
*E. faecium*-FL1	GAAAAAACAATAGAAGAATTAT	*sodA*	215	[29]
*E. faecium*-FL2	TGCTTTTTTGAATTCTTCTTTA			
*E. faecalis*-FM1	ACTTATGTGACTAACTTAACC	*sodA*	360	
*E. faecalis*-FM2	TAATGGTGAATCTTGGTTTGG			
vanA-A1	GGGAAAACGACAATTGC	*vanA*	732	[30]
vanA-A2	GTACAATGCGGCCGTTA			
vanB-B1	ATGGGAAGCCGATAGTC	*vanB*	635	
vanB-B2	GATTTCGTTCCTCGACC			
vanC1-C1	GGTATCAAGGAAACCTC	*vanC1*	822	
vanC1-C2	CTTCCGCCATCATAGCT			
vanC2/3-D1	CTCCTACGATTCTCTTG	*vanC2/C3*	438	
vanC2/3-D2	CGAGCAAGACCTTTAAG			

## Data Availability

No new data were created or analyzed in this study. Data sharing is not applicable to this article.

## References

[1] O’Driscoll T., Crank C.W. (2015). Vancomycin-resistant enterococcal infections: Epidemiology, clinical manifestations, and optimal management. Infect. Drug Resist..

[2] Tacconelli E., Carrara E., Savoldi A., Harbarth S., Mendelson M., Monnet D.L., Pulcini C., Kahlmeter G., Kluytmans J., Carmeli Y. (2018). WHO Pathogens Priority List Working Group. Discovery, research, and development of new antibiotics: The WHO priority list of antibiotic-resistant bacteria and tuberculosis. Lancet Infect. Dis..

[3] Rios R., Reyes J., Carvajal L.P., Rincon S., Panesso D., Echeverri A.M., Dinh A., Kolokotronis S.O., Narechania A., Tran T.T. (2020). Genomic epidemiology of vancomycin-resistant Enterococcus faecium (VREfm) in Latin America: Revisiting the global VRE population structure. Sci. Rep..

[4] Paladino J.A., Sunderlin J.L., Adelman M.H., Singer M.E., Schentag J.J. (2007). Observations on vancomycin use in U.S. hospitals. Am. J. Health Syst. Pharm..

[5] Kühn I., Iversen A., Finn M., Greko C., Burman L.G., Blanch A.R., Vilanova X., Manero A., Taylor H., Caplin J. (2005). Occurrence and relatedness of vancomycin-resistant enterococci in animals, humans, and the environment in different European regions. Appl. Environ. Microbiol..

[6] Shrestha S., Kharel S., Homagain S., Aryal R., Mishra S.K. (2021). Prevalence of vancomycin-resistant enterococci in Asia-A systematic review and meta-analysis. J. Clin. Pharm..

[7] Linden P.K. (2002). Treatment options for vancomycin-resistant enterococcal infections. Drugs.

[8] Raza T., Ullah S.R., Mehmood K., Andleeb S. (2018). Vancomycin resistant Enterococci: A brief review. J. Pak. Med. Assoc..

[9] Bassetti M., Poulakou G., Ruppe E., Bouza E., Van Hal S.J., Brink A. (2017). Antimicrobial resistance in the next 30 years, humankind, bugs and drugs: A visionary approach. Intensive Care Med..

[10] Metan G., Zarakolu P., Unal S. (2005). Rapid detection of antibacterial resistance in emerging Gram-positive cocci. J. Hosp. Infect..

[11] Endtz H.P., Van Den Braak N., Van Belkum A., Goessens W.H., Kref D., Stroebel A.B., Verbrugh H.A. (1998). Comparison of eight methods to detect vancomycin resistance in enterococci. J. Clin. Microbiol..

[12] Gorrie C., Higgs C., Carter G., Stinear T.P., Howden B. (2019). Genomics of vancomycin-resistant Enterococcus faecium. Microb. Genom.

[13] Ulu-Kilic A., Özhan E., Altun D., Perçin D., Güneş T., Alp E. (2016). Is it worth screening for vancomycin-resistant Enterococcus faecium colonization? Financial burden of screening in a developing country. Am. J. Infect. Control..

[14] Kramer T.S., Remschmidt C., Werner S., Behnke M., Schwab F., Werner G., Gastmeier P., Leistner R. (2018). The importance of adjusting for enterococcus species when assessing the burden of vancomycin resistance: A cohort study including over 1000 cases of enterococcal bloodstream infections. Antimicrob. Resist. Infect. Control.

[15] Kallstrom G., Doern C.D., Dunne W.M. (2010). Evaluation of a chromogenic agar under development to screen for VRE colonization. J. Clin. Microbiol..

[16] Teixeira L.M., Carvalho M.G.S., Shewmaker P.L., Facklam R.R., Versalovic J., Carroll K.C., Funke G., Jorgensen J.H., Landry M.L., Warnock D.W. (2011). Enterococcus. Manual of Clinical Microbiology.

[17] Adhikari L. (2010). High-level aminoglycoside resistance and reduced susceptibility to vancomycin in nosocomial enterococci. J. Glob. Infect. Dis..

[18] Zhou W., Zhou H., Sun Y., Gao S., Zhang Y., Cao X., Zhang Z., Shen H., Zhang C. (2020). Characterization of clinical enterococci isolates, focusing on the vancomycin-resistant enterococci in a tertiary hospital in China: Based on the data from 2013 to 2018. BMC Infect. Dis..

[19] Gold H.S. (2001). Vancomycin-resistant enterococci: Mechanisms and clinical observations. Clin. Infect. Dis..

[20] Arredondo-Alonso S., Top J., Corander J., Willems R.J.L., Schürch A.C. (2021). Mode and dynamics of vanA-type vancomycin resistance dissemination in Dutch hospitals. Genome Med..

[21] Hughes A., Ballard S., Sullivan S., Marshall C. (2019). An outbreak of vanA vancomycin-resistant Enterococcus faecium in a hospital with endemic vanB VRE. Infect. Dis. Health.

[22] Correa-Martinez C.L., Tönnies H., Froböse N.J., Mellmann A., Kampmeier S. (2020). Transmission of vancomycin-resistant enterococci in the hospital setting: Uncovering the patient-environment interplay. Microorganisms.

[23] Armin S., Zahedani S.S., Rahbar M., Azimi L. (2020). Prevalence and resistance profiles of vancomycin-resistant enterococcal isolates in Iran; An Eight-month Report from Nine Major Cities. Infect. Disord. Drug Targets.

[24] Dziri R., El Kara F., Barguellil F., Ouzari H.I., El Asli M.S., Klibi N. (2019). Vancomycin-resistant Enterococcus faecium in Tunisia: Emergence of novel clones. Microb. Drug Resist..

[25] Fioriti S., Simoni S., Caucci S., Morroni G., Ponzio E., Coccitto S.N., Brescini L., Cirioni O., Menzo S., Biavasco F. (2020). Trend of clinical vancomycin-resistant enterococci isolated in a regional Italian hospital from 2001 to 2018. Braz. J. Microbiol..

[26] Fujiya Y., Harada T., Sugawara Y., Akeda Y., Yasuda M., Masumi A., Hayashi J., Tanimura N., Tsujimoto Y., Shibata W. (2021). Transmission dynamics of a linear vanA-plasmid during a nosocomial multiclonal outbreak of vancomycin-resistant enterococci in a non-endemic area, Japan. Sci. Rep..

[27] Resende M., Caierão J., Prates J.G., Narvaez G.A., Dias C.A., d’Azevedo P.A. (2014). Emergence of vanA vancomycin-resistant Enterococcus faecium in a hospital in Porto Alegre, South Brazil. J. Infect. Dev. Ctries.

[28] Simner P.J., Adam H., Baxter M., McCracken M., Golding G., Karlowsky J.A., Nichol K., Lagacé-Wiens P., Gilmour M.W., Canadian Antimicrobial Resistance Alliance (CARA) (2015). Epidemiology of vancomycin-resistant enterococci in Canadian hospitals (CANWARD study, 2007 to 2013). Antimicrob. Agents Chemother..

[29] Jackson C.R., Fedorka-Cray P.J., Barrett J.B. (2004). Use of a genus- and species-specific multiplex PCR for identification of enterococci. J. Clin. Microbiol..

[30] Pérez-Hernández X., Méndez-Alvarez S., Claverie-Martín F. (2002). A PCR assay for rapid detection of vancomycin-resistant enterococci. Diagn. Microbiol. Infect. Dis..

[31] Clinical and Laboratory Standards Institute (2021). Performance Standards for Antimicrobial Susceptibility Testing.

[32] McHugh M.L. (2012). Interrater reliability: The kappa statistic. Biochem. Med..

